# Impacts of visual impairment on pragmatic impairment: A systematic review and meta-analysis

**DOI:** 10.1371/journal.pone.0294326

**Published:** 2023-12-08

**Authors:** Cheng Lulu, Hong Xie, Peng Wang, Ting Zhang

**Affiliations:** 1 School of Foreign Studies, China University of Petroleum (East China), Qingdao, China; 2 Shanghai Center for Research in English Language Education, Shanghai International Studies University, Shanghai, China; 3 School of International Education, Guizhou Normal University, Guiyang, China; 4 College of Engineering, Beijing University, Beijing, China; Federal University of Paraiba, BRAZIL

## Abstract

**Background:**

Consideration for patients with visual impairment, from low vision to blindness, is an important part of building a barrier-free society. Some authors have elaborated that visual impairment can indeed lead to delayed development in theory of mind, thereby causing pragmatic knowledge deficiency. Verifying whether those with eye conditions have pragmatic impairment is an essential way for their clinical evaluation, intervention and rehabilitation.

**Objective:**

We primarily carry out a meta-analysis of visual impairment from low vision to blindness and pragmatic impairment in people with low vision or blindness to verify visual impairment may cause pragmatic impairment.

**Data sources:**

Electronic databases Pubmed, Medline, MesH, Psychinfo, Ovid, EBSCO and CNKI and the reference sections of previous reviews.

**Study eligibility criteria:**

Studies were included when they built on primary data from clinical questionnaire surveys or field trials anywhere in the world, and when they reported impacts of visual impairment on social cognition, communication, skills, behavior and intelligence. In total, 25 original studies were included, in which 25735 people were evaluated.

**Results:**

Statistically, visual impairments and pragmatic impairment exist correlation due to the significant p value(*p* = 0.0005 < 0.05) in group and the subgroup sorted in the light of 18 years old (*p* < 0.0001 and *p* = 0.003 < 0.05). Psychologically, because people with visual impairment can not normally get non-verbal information, they can not get a complete pragmatic knowledge system. Pragmatic knowledge deficiency leads to abnormal in executive functions and development delay from the perspective of theory of mind, inducing pragmatic impairment. Therefore, visual impairment has an impact on pragmatic impairment.

**Conclusion:**

The meta-analysis reveals robust evidence on the relationship of vision impairment and pragmatic impairment in children or adults. Such evidence may help to gradually improve the clinical evaluation, intervention and rehabilitation of these people.

## Introduction

Recently, with the development of Internet of things, blockchain, artificial intelligence and metaverse, the construction of barrier-free society accelerates [1]. And technology empowers life, further deepening the depth and breadth of the application of digital intelligence technology in interpersonal communication. Meanwhile, according to the Global Report on Traditional and Complementary Medicine launched by the World Health Organization in 2019, pragmatic communication problems of people with visual impairment from low vision to blindness have gained enormous attentions [2]. Particular, such eye conditions result in pragmatic impairment, leading to significant adverse effects in their social communication.

Pragmatic impairment usually refer to “semantic-pragmatic language disorder” or “pragmatic language disorders” or “pragmatic language difficulties” [3–5]. The previous studies on visual impairment and pragmatic impairment mainly focuses on the following two aspects: (a) Language comprehension impairment. Children with congenital visual impairment can trigger delayed psychological development, especially in terms of second theory of mind, awareness of other people’s belief, and non-verbal language like irony or rhetoric. In addition, they are unable to understand the intention of verbal communication in a timely and accurate manner [6–9]. (b) Social behavior impairment. James et al. (2007) [10] and Jeremy et al. (2010) [11] clinically found that with the deepening of the degree of visual impairment, children or adults were difficult to express their behavior. And their social function decreased, and social communication difficulties further manifested. Specifically, their reception of social signals such as facial expressions, gestures and body posture, is usually low resolution ratio, and is limited by some environmental factors like push and squeeze or surrounding noise [12–16].

Language comprehension and social communication is a part of pragmatic competence performance [17]. Hence, previous researches verified that individuals with low vision or blindness suffer from mild to severe pragmatic impairment, resulting in incoherent narration and non-cooperative social interaction [18]. The coherent narration and fluent cooperation is the driver of executive functions and development outcome of the mind theory [19–21]. Hence, people with visual impairment have abnormal executive functions and delay the mind theory development [7, 22]. In other words, they can not speculate on their own and others’ psychological states like intentions, wishes, beliefs, motivations and emotions by a complete knowledge system, and make relationship prediction and interpretation of others’ social behaviors, which includes planning, inhibition, coordination and control of activity sequence, working memory and psychological flexibility [23–25].

Some studies consider that visual impairments have an impact on pragmatic impairment [6–25]. If there are contradictions for the result, a meta-analysis will help to understand the level of evidence between their relationships evaluated, since it seeks to summarize the results found. Nevertheless, rare meta-analysis is conducted to support this conclusion. Thus, we went beyond average impacts at micro level and used meta-regressions by the meta analysis to explain impact heterogeneity and test for possible biases. Our study provides the summary evidences for the assumption, visual impairment in minors and adults may lead to pragmatic impairment on most even all social activities given the significant *p* value.

## Materials and method

We performed this study according to Preferred Reporting Items for Systematic Reviews and Meta-Analysis (PRISMA) guidelines (**S1 Checklist**). Subjects in the included articles and participants who triggered the inspiration for our article have given written informed consent to publish these case details. During the whole research, Hong Xie and Peng Wang carry out the data search blindly and separately, while Lulu Cheng monitors and summarizes the included data and their quality. All data were anonymously analyzed, and this study was reviewed and allowed by the Academic Ethics Review Committee in School of Foreign Studies of China University of Petroleum (East China) (IRB) in China.

### Literature search and selection criteria

We searched the electronic databases Pubmed, Medline, MesH, Psychinfo, Ovid, EBSCO, WOS, Ovid, HMIC, ERIC and CNKI, the reference sections of previous reviews from their earliest entries to December 20, 2022. The exact electronic search strategy and a full description are offered in **S1 File**.

Studies published in English that meets the following criteria is included: (1) the study’s subjects should be over 3 years old and had signed the informed consent form under the supervision of parents; (2) their subjects are either mild to severe visual impairment with eyes’ degree about 0.1 ~ 0.8 or blindness. After medical treatment, they are still unable (or very difficult) to make accurate visual recognition to external things; (3) their subjects do not have basic disease, drug allergy history and are not gravida; (4) these studies aimed to explore visual impairment and pragmatic impairment like social cognition, communication, skills, behavior and intelligence in people from low vision to blindness in minors or adults. And there are no interests from authors and the experimental process and results are clear.

Studies were excluded if: (1) their research topics are not related to pragmatics; (2) methods and subjects are not in line with experimental specifications; (3) there are some interests from authors; (4) visual impairment with eyes’ degree over 0 ~ 0.8; (5) there are some faultiness in research results; (6) research process is unclear. The detailed inclusion or exclusion criteria and flowchart are provided in **S2 File.**

**Fig 1:**
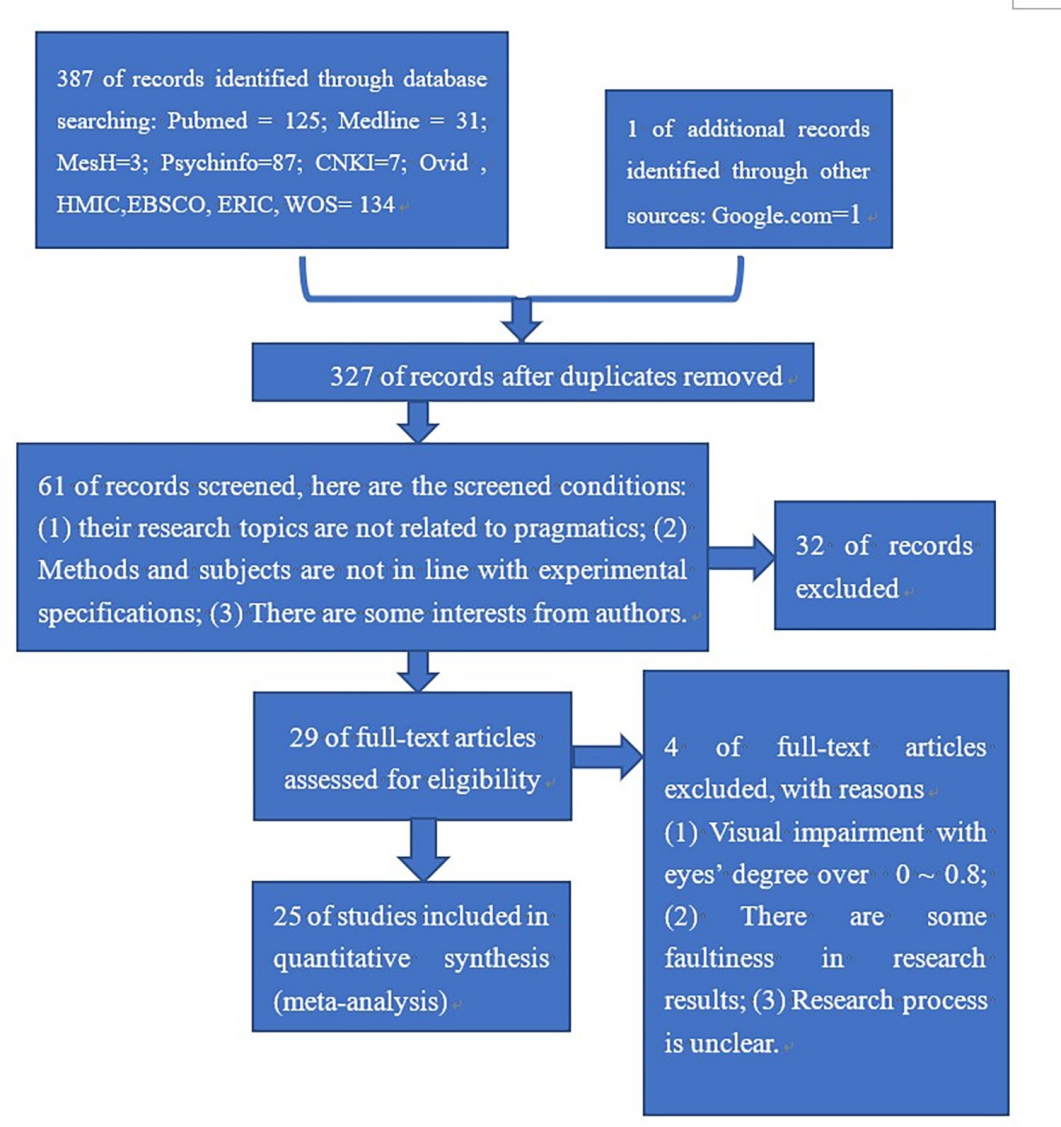
Flow chart of study selection process.

The electronic search yielded 387 articles and 1 book in the study. After removing duplicates, 327 articles were left. We reviewed their titles, abstracts and references and eliminated any articles that clearly fell outside our inclusion criteria. If there was any doubt that can not be solved by researchers, the article was directly excluded. Combined, this process retained 29 articles. Subsequently, three researchers (Hong Xie, Lulu Cheng and Peng Wang) examined the full text of each article and made independent judgments as to whether the article met our inclusion or exclusion criteria. Disagreements were settled by face-to-face discussion until a consensus judgment was reached. Finally, 25 articles met our inclusion and exclusion criteria. The selection process is illustrated in **Fig 1**.

### Risk of bias assessment

The method of risk of bias assessment is based on random allocation and allocation concealment (selection bias), personnel and participants (performance bias), result evaluation (detection bias), incomplete result data (natural attrition bias), selection of reported results (reporting bias) and sources (other biases) to display low, high and unclear bias risks for each included articles [26]. So according to its principle and practical steps, the 25 included studies in our study was evaluated by using the Cochrane collaboration’s tool, RoB2, which is a domain-based evaluation tool in randomized trial [25]. Two independent researchers (Hong Xie and Peng Wang) separately assessed the eligibility and assessed the risk bias of the included studies. Any disagreement in screening the articles was resolved through discussion between these two researchers, with adjudication by the third researcher (Lulu Cheng) if disagreements persisted.

According to the Cochrane principles with the combination of reviewers’ answers in our study, the risk of bias in each field may be divided into three levels: “low risk of bias”, “some concerns” and “high risk of bias” [27]. If the risk of bias assessment result in all fields is “low risk”, then the overall risk of bias is “low risk”. If the risk of bias evaluation result of some fields is “with certain risk” and there is no “high risk” field, then the overall risk of bias is “some concerns” [27]. As long as the assessment result of risk of bias in one field is “high risk”, the overall risk of bias is “high risk” [25, 27, 28].

### Data analysis

First of all, we conducted quality risk assessment on the included articles [29–46] by Review Manager 5.4 software to avoid the impact of high-risk dimensions on the study results [47].

Next, we assumed that patients with visual impairment will trigger pragmatic impairment. And the visual impairment was considered as the independent variable, while the pragmatic impairment was considered as the dependent variable. Duet to different samples characteristic in our included studies, we standardized mean difference in outcomes between the experimental and control groups to judge their risk bias. Meanwhile, we chose to use a random-effects model to summarize across studies. A random effects model assumes that the true intervention effect size varies depending on characteristics of the population studied or intervention employed.

A random-effects model is more conservative than a fixed-effects model because it produces a wider confidence interval for the summary effect size [48]. Therefore, we chose the *p* value in the Random Effect test to identify whether visual impairment will lead to pragmatic impairment. Meanwhile, because the age of different study samples is various, we used 18 years old as the cut-off point to conduct subgroup analysis after the random effect.

## Discussion and results

### Included studies characteristics

For the included 25 studies from 1995 to 2022 (Table 1), they are all come from SSCI, SCI, SCIE or ESCI publications in Pubmed, Medline and WOS websites. For instance, *Journal of autism and developmental disorders*, *Journal of visual impairment & blindness*, *Journal of child psychology and psychiatry*, *Brain and language*, *Psychiatry and clinical neurosciences*, *Autism* and so forth. Hence, in publications and research field, they have good representative.

**Table 1:** Included studies characteristics.

Author	Year	N	Age	Pragmatic Impairment Performance	Weight	Risk Ratio	Z-score
						Risk Ratio \ M-H, Random, 95% CI	
Anthony et al.	1999	18	6–13	can not understand non-literal language and advanced theory of mind	4.10%	1.40 [0.71, 2.77]	-0.477
Anthony et al.	2010	16	7–15	language impairment	4.00%	1.17 [0.53, 2.57]	0.147
Brunes et al.	2019	15620	7–18	abnormally communicate	4.40%	40.17 [33.45, 48.25]	4.976
Carretti et al.	2022	96	3–18	behavior like autism	4.30%	2.43 [1.51, 3.91]	0.671
Elizabeth et al.	2009	85	< 18	social communication difficulties	3.90%	6.98 [2.66, 18.27]	1.894
Glatz et al.	2022	7715	3–18	communication and cooperation difficulties	4.40%	6.00 [4.90, 7.34]	1.616
Habib et al.	2018	80	3–33	behavior like autism	3.80%	4.00 [1.47, 10.92]	1.061
Haegele et al.	2018	12	3–9	abnormal executive functions	3.40%	4.33 [1.03, 18.17]	1.119
Heppe et al.	2020	76	50 or so	intellectual disabilities can not develop completely with the increase of age	4.00%	8.98 [3.86, 20.89]	2.261
James et al.	2006	8	3–8	behavior like autism	4.20%	0.94 [0.50, 1.78]	0.243
Jeremy et al.	2010	83	3–8	individuals with visual impairment exist autism	4.30%	1.01 [0.75, 1.34]	0.257
Jesper	2014	138	< 15	communication skills lag behind common children	4.30%	2.29 [1.55, 3.37]	0.571
Judith et al.	2012	48	4–11	social understanding development lags behind common children	4.40%	1.00 [0.89, 1.13]	0.242
Linda et al.	1998	32	9–12	problems with social cognition	3.70%	3.00 [0.99, 9.08]	0.712
Lisa et al.	2011	41	13–19	can not understand correctly semantic relations	4.40%	0.74 [0.56, 0.96]	0.169
Lourens et al.	2016	30	7–15	conservation difficulties	3.30%	10.33 [2.25, 47.53]	2.321
McAlpine et al.	1995	31	>18	have impact on life satisfaction	3.90%	0.89 [0.34, 2.31]	0.211
Mukaddes et al.	2007	257	>18	education difficulties	4.40%	1.31 [1.13, 1.52]	0.302
Nawoja	2015	104	15–22	social participation difficulties	4.30%	2.71 [1.68, 4.37]	0.604
Pearl	2010	102	13–18	have impact on life quality	4.30%	1.00 [0.63, 1.59]	0.211
Rachel et al.	1997	43	>18	pragmatic development vary	3.50%	5.94 [1.54, 22.84]	1.216
Ralejoe	2021	10	18–33	physical education difficulties	2.30%	5.67 [0.45, 71.51]	1.063
Splunder et al.	2006	850	16–23	people are trouble in inclusion to these people	4.40%	0.99 [0.88, 1.13]	0.166
Timothy et al.	2005	160	>18	low psychological well-being and life quality	4.40%	1.88 [1.51, 2.34]	0.293
Urbaniak-Olejnik et al.	2022	80	18–24	hard to keep body balance	3.70%	15.00 [4.98, 45.22]	2.088
					-	Average Z-score	0.958

Furthermore, we employed confidence interval (CI) to justice whether there is significant difference in included articles because participants in some included articles are significantly more than others. Statistically, 95%CI refers to the risk ratio will occur in the area with a possibility of 95% [49]. For example, as can be seen from Table 1, the article written by Anthony et al. (1999) have 95% CI from 0.71 to 2.77, meaning that in this experiment, visually impaired patients are about 3 times (2.77 / 0.71) than non-visually impaired patients in terms of experiencing pragmatic impairment. According to this statistical step, we can find that whether studies with more than 100 experimental samples or lower than 100 experimental samples, their 95% CI difference between the minimum value and maximum value are equal to or greater than 1 time. Thus, the number of samples in the included articles has no significant difference, and has no impact on their research results [50]. Additionally, the experimental sample weight of different studies is centered around the median, 4.20%, and roughly evenly distributed on both sides. And this figure is closed to their average, 4.00%. The comparation indicates that the two figures are complementary to each other and each included article demonstrates similar contribution in our study. The average Z-score is 0.958, less than 1.000, indicating that each included article has sound accuracy and interpretability [51]. Combined, the quality of the included studies is similar and holds excellent validity in research time, experimental subjects and experimental specifications.

### Risk of bias

**Fig 2:**
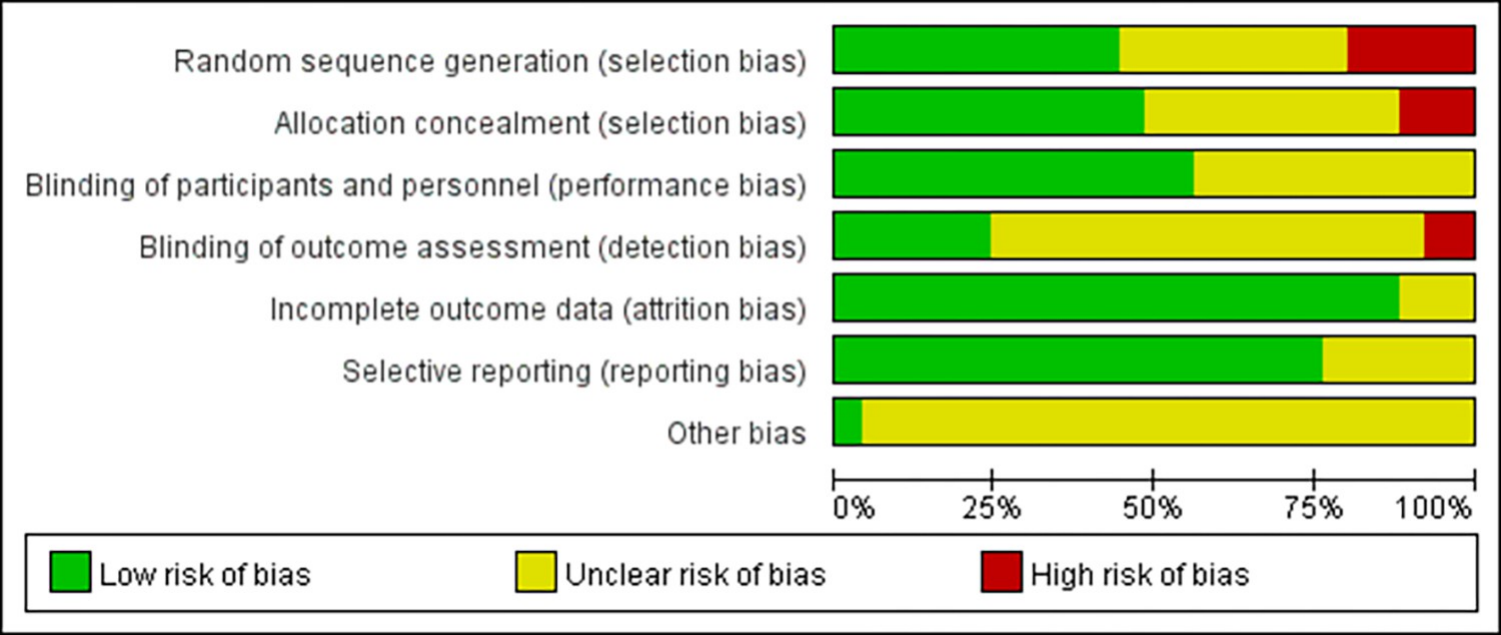
Risk of bias.

Evidently, risk of bias assessment in the included articles in our meta analysis is comparatively qualified because the average ratio of low risk of bias is over 50%, while the total ratio of high risk of bias is roughly 10% (**Fig 2**). Some studies were assessed to unclear risk of bias because they did not provide information about randomization. Therefore, we can draw the following two results.

### Visual impairment may cause pragmatic impairment

**Fig 3:**
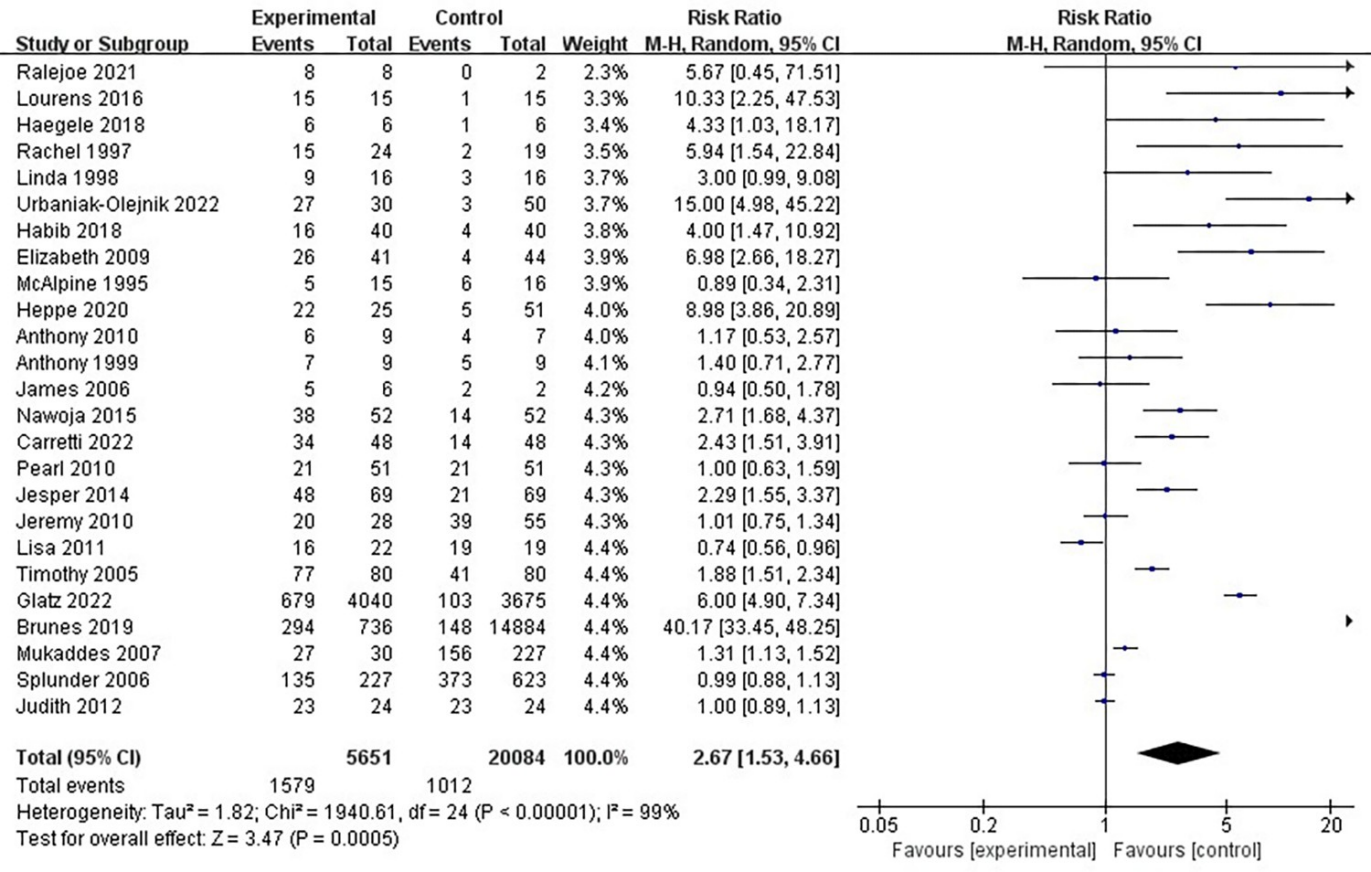
Forest plot of the random effect of the relationship between visual impairment and pragmatic impairment.

**Fig 3** displays the *p* value in the test for overall effect is 0.0005, which is less than 0.05. And the I square is 99%, manifesting that the forest plot generated from random effect is reasonable and its results have high interpretability [52]. Hence, our assumption is statistically significant, that is, the relationship of visual impairment and pragmatic impairment significantly exists. At the same time, the diamond located in approximately 1–5 odds ratio (in the range of 1–15), which does not intersect with invalid line, further supporting this conclusion statistically [48]. Visually impaired population cannot fully leverage their vision as a major medium to communicate.

With limited visual information receiving, individuals with visual impairment rely heavily on their auditory sensory channel regions like thalamus and temporal lobe to receive and comprehend information in pragmatic information reception [53]. Nevertheless, individuals with visual impairment, whether due to congenital conditions, acquired injuries, or progressive eye diseases, the thalamus may undergo certain changes and adaptations [7, 9, 54]. And the temporal lobe is also crucial for language comprehension, and visual impairment may influence how individuals with this condition process and understand language-related information [55]. Those factors about information processing in auditory channels may result in pragmatic impairment. Furthermore, auditory information transmission passes thalamus, temporal lobe, and Broca’s area controlling speech communication including nucleus basalis magnocellularis cholinergic system [56], which plays an essential role in attention performance, executive functions of discourse comprehension and theory of mind development [57–59].

Moreover, psychological factors including social isolation, low self-esteem and self-confidence, anxiety and stress, communication apprehension can play a significant role in pragmatic impairment experienced by the visually impaired population [22, 36]. For instance, visually impaired individuals may develop communication apprehension, fearing potential misunderstandings or misinterpretations during interactions. This fear can lead to avoidance of social communication, perpetuating pragmatic impairment. Thus, interventions focused on building self-confidence, reducing anxiety, fostering social support networks, and providing training in effective social communication can be beneficial in improving pragmatic language skills in this population.

Additionally, researchers have shown that there is an increased likelihood of individuals, from birth or from an early childhood, with visual impairment also having autism [60–63]. Lipton (1979) named those blind children with a stereotypical and repetitive behavior as “blindism” [64]. While Wrzesinska (2017) put that the deficiency of sensory and social stimuli might be responsible for the development of autistic-like behaviors. So visual impairment in children made their psycho-social development slower and might account for development of autistic-like behaviors as well. Although no explicit confirmation showed that some visual impairment was a factor of determining autism, individuals with visual impairment and autism have weathered pragmatic impairment, mostly with reference to their relations with other people and emotional expression due to a delay in more advanced theory of mind understanding [3, 7, 8, 19–21]. Therefore, with the combination of statistical results in this study and current literature findings, people with visual impairment from low vision to blindness may cause pragmatic problems.

### Age do not affect pragmatic impairment occurrence

**Fig 4:**
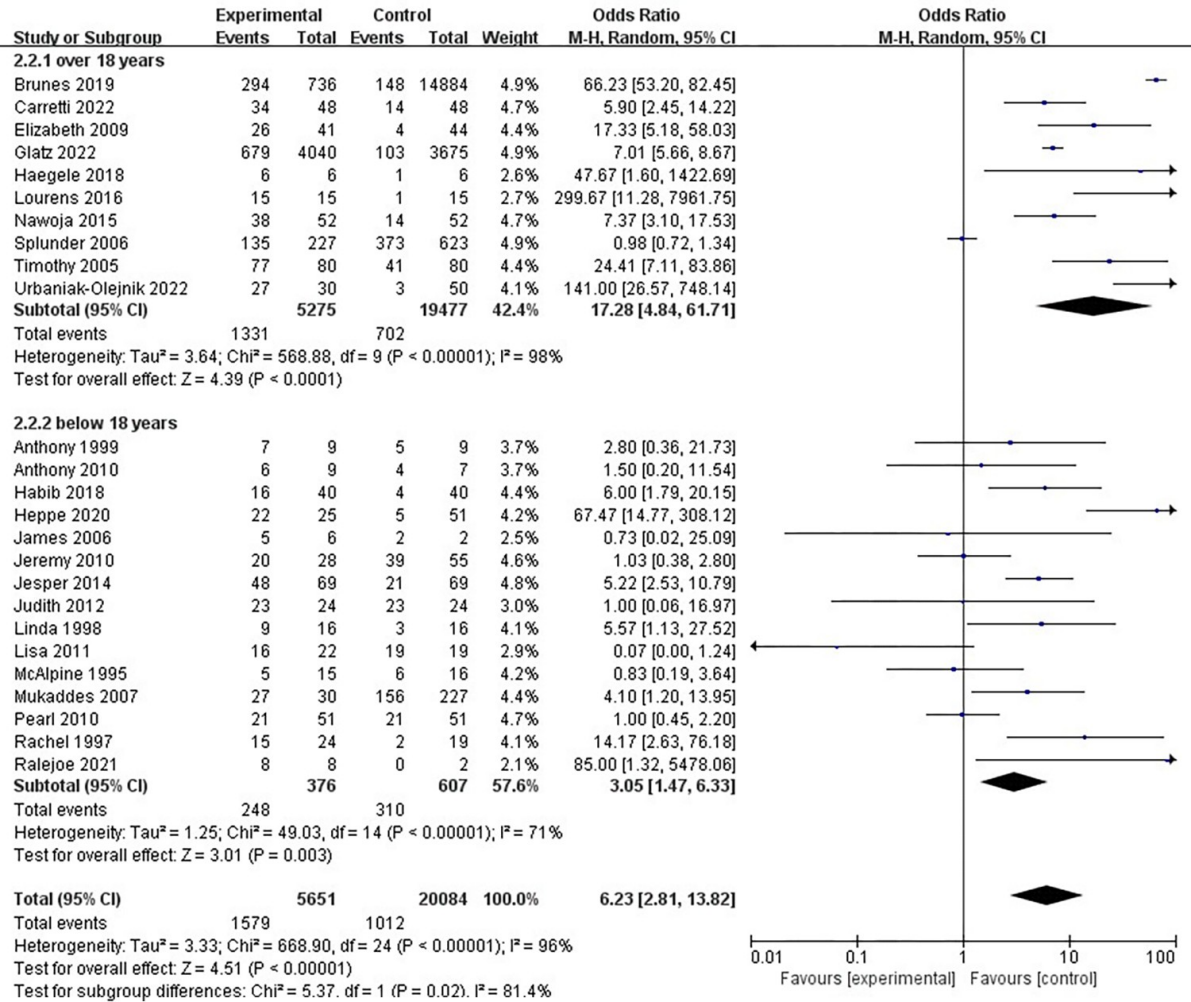
Subgroup forest plot of the random effect of the relationship between age and pragmatic impairment.

Some studies consider that age plays an essential role in visual impairment, particularly for the children, and the adults given their different eyeball changes in terms of structure and functions [57, 65, 66]. So it is necessary to conduct a subgroup analysis for adults and minors. As can be seen from **Fig 4**, samples were divided into over 18 years old and below 18 years old. On the one hand, the *p* value is significant. The former *p* < 0.0001, and the latter *p =* 0.003 < 0.05, which are obviously significant in the relation assumption of age and pragmatic impairment. Additionally, because some participants in included articles were just 18 years old, which is difficult to distinguish their subgroup, we distribute them evenly between two groups. The distribution may result in the slightly high of I square in the two subgroups, with the value of 98% and 71%, respectively. However, compared with 96% I square (P<0.0001) after their combination, the two figure still have interpretability and can infer a reliable result [52].

There are 5275 people over 18 years old in experimental group, while there are 376 people below 18 years old. The two number are lower than that of control group and the diamond is also in the left side (in the range of 1–15), indicating subgroup analysis result is in line with the superior analysis result [67]. Visual impairment occurs at different ages, including congenital visual impairment and acquired visual impairment. Congenital visual impairment accompanies the whole process of language acquisition, especially the development and maturity of pragmatic competence [4, 12, 23]. Acquired visual impairment is closely related to patients’ psychological endurance and mental status, and plays an important role in the multimodal information reception process in social interaction. Furthermore, pragmatic competence is a vital ability for people to communicate with each other at any age. Therefore, by and large, pragmatic impairment is independent of the age among individuals from low vision to blindness.

## Conclusion

This analysis from the included articles with relatively low risk of bias as a whole provides evidence for the assumption that visual impairment may cause pragmatic impairment statistically and psychologically. The argumentation also provides a theoretical basis for further study of discourse pragmatic impairment of people with visual impairment or autism spectrum disorder and visual impairment concurrently. Moreover, these results also offer a theoretical dimension for clinical identification and rehabilitation of individuals with visual impairment. However, some limitations can be seen in our study. On the one hand, studies carried out before 1995 are ignored and experimental subjects below 3 years old are excluded, resulting in a limited research scope in year and age. On the other hand, the research results are limited by the research quality of the included articles. Whether articles with different quality have impacts on research results also deserves to discuss in depth. Therefore, further research could be focused on some detailed areas, for instance, the different quality influence of the included literature to research results, the features of visual impairment and pragmatic impairment in different ages and different social communication, the brain cognition process of people with visual impairment and pragmatic impairment like the phonetic-vocabulary processing mechanism and semantic conversion process.

## Supporting information

S1 Checklist PRISMA checklist. (PDF)

S1 File Electronic search strategy. (PDF)

S2 File Inclusion/exclusion criteria and flow. (PDF)

## References

[1] Yaroshenko O M, Anisimova H V, Koliesnik T V, et al. National strategy for a barrier-free environment: problems, tolerance and implementation. International social work, 2022: 00208728221126002. 10.1177/00208728221126.

[2] World Health Organization. WHO global report on traditional and complementary medicine 2019. Geneva: World Health Organization, 2019.

[3] Martin I, McDonald S. Weak coherence, no theory of mind, or executive dysfunction? Solving the puzzle of pragmatic language disorders. Brain and language, 2003, 85(3): 451–466. doi: 10.1016/s0093-934x(03)00070-1 12744957

[4] Ryder N, Leinonen E, Schulz J. Cognitive approach to assessing pragmatic language comprehension in children with specific language impairment. International journal of language & communication disorders, 2008, 43(4): 427–447. doi: 10.1080/13682820701633207 18584419

[5] Perkins M R. Pragmatic impairment. The handbook of language and speech disorders, 2010: 227–246. 10.1002/9781119606987.ch10.

[6] Van Splunder J, Stilma J S, Bernsen R M D, et al. Prevalence of visual impairment in adults with intellectual disabilities in the Netherlands: cross-sectional study. Eye, 2006, 20(9): 1004–1010. doi: 10.1038/sj.eye.6702059 16151486

[7] Judith P. Mathijs P. J. Steenbergen B. Pragmatic abilities in children with congenital visual impairment: an exploration of non-literal language and advanced theory of mind understanding. Journal of autism and developmental disorders, 2012, 42: 2440–2449. doi: 10.1007/s10803-012-1500-5 22437442 PMC3474910

[8] Nawoja M. The associative structure of the mental lexicon: hierarchical semantic relations in the minds of blind and sighted language users. Psychology of language and communication, 2015, 19(1): 1–18. 10.1515/plc-2015-0001.

[9] Chokron S, Kovarski K, Zalla T, et al. The inter-relationships between cerebral visual impairment, autism and intellectual disability. Neuroscience & biobehavioral reviews, 2020, 114: 201–210. doi: 10.1016/j.neubiorev.2020.04.008 32298709

[10] James D M, Stojanovik V. Communication skills in blind children: a preliminary investigation. Child: care, health and development, 2007, 33(1): 4–10. doi: 10.1111/j.1365-2214.2006.00621.x 17181747

[11] Jeremy R P, Naomi J D, Lara M S, et al. Social Communication difficulties and autism spectrum disorder in young children with optic nerve hypoplasia and / or septo-optic dysplasia. Developmental medicine & child neurology, 2010, 52(10): 917–921. 10.1111/j.1469-8749.2010.03664.x. 20370811

[12] Linda P, Dewart H, Brockbank M. Social cognition in children with visual impairments. Journal of visual impairment & blindness, 1998, 92(11): 754–768. 10.1177/0145482x9809201104.

[13] Haegele J A, Kirk T N, Zhu X. Self-efficacy and physical activity among adults with visual impairments. Disability and health journal, 2018a, 11(2): 324–329. doi: 10.1016/j.dhjo.2017.10.012 29126897

[14] Haegele J A, Zhu X, Kirk T N. Weekday physical activity and health-related fitness of youths with visual impairments and those with autism spectrum disorder and visual impairments. Journal of visual impairment & blindness, 2018b, 112(4): 372–384. 10.1177/0145482x1811200404.

[15] Brunes A, B. Hansen M Heir T. Loneliness among adults with visual impairment: prevalence, associated factors, and relationship to life satisfaction. Health and quality of life outcomes, 2019, 17: 1–7. 10.1186/s12955-019-1096-y. 30709406 PMC6359849

[16] Qiu S, An P, Hu J, et al. Understanding visually impaired people’s experiences of social signal perception in face-to-face communication. Universal access in the information society, 2020, 19: 873–890. 10.1007/s10209-019-00698-3.

[17] Perkins M. Pragmatic impairment. Cambridge: Cambridge University Press. 2007.

[18] Cummings L. Clinical pragmatics. Cambridge: Cambridge University Press, 2009.

[19] Marocchini E. Impairment or difference? The case of theory of mind abilities and pragmatic competence in the autism spectrum. Applied psycholinguistics, 2023: 1–19. 10.1017/s0142716423000024.

[20] Nguyen T N, Gonzalez C. Theory of mind from observation in cognitive models and humans. Topics in cognitive science, 2022 (4): 665–686. doi: 10.1111/tops.12553 34165919

[21] Hamilton J, Radlak B, Morris P G, et al. Theory of mind and executive functioning following stroke. Archives of clinical neuropsychology, 2017, 32(5): 507–518. doi: 10.1093/arclin/acx035 28453602

[22] Krisi M, Nagar R, Knoll N. Psychological factors involved in the acquisition of a foreign language among students with visual impairments. British journal of visual impairment, 2022 (2): 196–208. 10.1177/0264619620961806.

[23] Cummings L. Cognitive aspects of pragmatic disorders. Research in clinical pragmatics, 2017: 587–616. 10.1007/978-3-319-47489-2_22.

[24] Cummings L. Research in Clinical Pragmatics. Nottingham: Springer, 2017.

[25] Minozzi S, Dwan K, Borrelli F, et al. Reliability of the revised Cochrane risk-of-bias tool for randomised trials (RoB2) improved with the use of implementation instruction. Journal of clinical epidemiology, 2022, 141: 99–105. doi: 10.1016/j.jclinepi.2021.09.021 34537386

[26] Page MJ, Higgins J, Sambunjak D, et al. Module 5: introduction to study quality and risk of bias. in: cochrane interactive learning: conducting an intervention review. cochrane, 2017. Available from https://training.cochrane.org/interactivelearning/module-5-introduction-study-quality-and-risk-bias.

[27] Higgins J P T, Sterne J A C, Savovic J, et al. A revised tool for assessing risk of bias in randomized trials. Cochrane database of systematic reviews, 2016, 10(1): 29–31. https://methods.cochrane.org/bias/resources.

[28] Sterne J A C, Hernán M A, Reeves B C, et al. ROBINS-I: a tool for assessing risk of bias in non-randomised studies of interventions. Bmj, 2016, 355. doi: 10.1136/bmj.i4919 27733354 PMC5062054

[29] Anthony L, Hobson R P. Reversible autism among congenitally blind children? a controlled follow‐up study. Journal of child psychology and psychiatry, 2010, 51(11): 1235–1241. doi: 10.1111/j.1469-7610.2010.02274.x 20584101

[30] Lourens H, Swartz L. Experiences of visually impaired students in higher education: bodily perspectives on inclusive education. Disability & society, 2016, 31(2): 240–251. doi: 10.1080/09687599.2016.1158092 27917028 PMC5130161

[31] Heppe E C M, Willemen A M, Kef S, et al. Improving social participation of adolescents with a visual impairment with community-based mentoring: results from a randomized controlled trial. Disability and rehabilitation, 2020, 42(22): 3215–3226. doi: 10.1080/09638288.2019.1589587 31066313

[32] Habib F, Irshad E. Impact of Visual Impairment on Quality of Life among Adolescents. FWU Journal of Social Sciences, 2018, 12(1): 149–155. 10.1177/0264619617737123.

[33] Glatz M, Riedl R, Glatz W, et al. Blindness and visual impairment in central Europe. Plos one, 2022, 17(1): 1–14. doi: 10.1371/journal.pone.0261897 35025896 PMC8758103

[34] Haegele J A, Kirk T N. Experiences in physical education: exploring the intersection of visual impairment and maleness. Adapted physical activity quarterly, 2018, 35(2): 196–213. doi: 10.1123/apaq.2017-0132 29529866

[35] Ralejoe M. A study to understand the inclusion of learners with and without visual impairment in a secondary school in Lesotho. South African journal of education, 2021, 41(1): 1–12. 10.15700/saje.v41n1a1746.

[36] Carretti G, Mirandola D, Sgambati E, et al. Survey on psychological well-being and quality of life in visually impaired individuals: dancesport vs. other sound input-based sports. International journal of environmental research and public health, 2022, 19(8): 4438. doi: 10.3390/ijerph19084438 35457304 PMC9024582

[37] Urbaniak-Olejnik M, Loba W, Stieler O, et al. Body balance analysis in the visually impaired individuals aged 18–24 years. International journal of environmental research and public health, 2022, 19(21): 14383. doi: 10.3390/ijerph192114383 36361259 PMC9654500

[38] Anthony L, Peter H R, Brown R. Autism and congenital blindness. Journal of autism and developmental disorders, 1999, 29: 45–56. doi: 10.1023/a:1025918616111 10097994

[39] Elizabeth M E, Helen G E, David B E, et al. Vision in children and adolescents with autistic spectrum disorder: evidence for reduced convergence. Journal of autism and developmental disorders, 2009, 39(7): 965–975. doi: 10.1007/s10803-009-0705-8 19224351

[40] Jesper D. Symptoms of autism among children with congenital deafblindness. Journal of autism and developmental disorders, 2014, 44: 1095–1102. doi: 10.1007/s10803-013-1967-8 24127166

[41] Lisa M R, Cornish K M, Fombonne E. Diagnostic differentiation of autism spectrum disorders and pragmatic language impairment. Journal of autism and developmental disorders, 2011, 41: 1694–1704. doi: 10.1007/s10803-011-1196-y 21416199

[42] McAlpine L M, Moore C L. The development of social understanding in children with visual impairments. Journal of visual impairment & blindness, 1995, 89(4): 349–358. 10.1177/0145482x9508900408.

[43] Klümper W, Qaim M. A meta-analysis of the impacts of genetically modified crops. Plos one, 2014, 9(11): e111629. doi: 10.1371/journal.pone.0111629 25365303 PMC4218791

[44] Mukaddes N M, Kilincaslan A, Kucukyazici G, et al. Autism in visually impaired individuals. Psychiatry and clinical neurosciences, 2007, 61(1): 39–44. doi: 10.1111/j.1440-1819.2007.01608.x 17239037

[45] Pearl E. Visual impairment, verbal humor, and conservation. The Journal of genetic psychology, 1986, 147(1): 107–111. doi: 10.1080/00221325.1986.9914485 3723119

[46] Rachel B, Hobson R P, Lee A, et al. Are there “autistic‐like” features in congenitally blind children?. Journal of child psychology and psychiatry, 1997, 38(6): 693–703. doi: 10.1111/j.1469-7610.1997.tb01696.x 9315979

[47] Timothy S H, Tina L, et al. Parker. Autistic‐like behavior in CHARGE syndrome. American journal of medical genetics, 2005, 133(3): 257–261. 10.1002/ajmg.a.30545. 15637726

[48] DerSimonian R, Laird N. Meta-analysis in clinical trials. Controlled clinical trials, 1986, 7(3): 177–188. doi: 10.1016/0197-2456(86)90046-2 3802833

[49] Neyman J. Outline of a theory of statistical estimation based on the classical theory of probability. Philosophical transactions of the royal society A. 1937, 236 (767): 333–380. 10.1525/9780520327016-022.

[50] Haase M, Bellomo R, Devarajan P, et al. Accuracy of neutrophil gelatinase-associated lipocalin in diagnosis and prognosis in acute kidney injury: a systematic review and meta-analysis. American journal of kidney diseases, 2009, 54(6): 1012–1024. 10.1053/j.ajkd.2009.07.020. 19850388

[51] Curtis A E, Smith T A, Ziganshin B A, et al. The mystery of the Z-score. Aorta, 2016, 4(04): 124–130. doi: 10.12945/j.aorta.2016.16.014 28097194 PMC5217729

[52] Higgins J P T, Thompson S G, Deeks J, et al. Measuring inconsistency in meta-analyses. British medical journal. 2003, 327(7414): 557–560. doi: 10.1136/bmj.327.7414.557 12958120 PMC192859

[53] Gao S, Wang Y, Gao X, et al. Visual and auditory brain–computer interfaces. IEEE transactions on biomedical engineering, 2014, 61(5): 1436–1447. doi: 10.1109/TBME.2014.2300164 24759277

[54] Kelly C, Meyer J, Hanks V, et al. Neurorehabilitation for an individual with bilateral thalamic stroke and preexisting visual impairment presenting with impaired use of sensory cues: A case report. Physiotherapy theory and practice, 2021, 37(10): 1139–1145. doi: 10.1080/09593985.2019.1683920 31657267

[55] Girardi-Schappo M, Fadaie F, Lee H M, et al. Altered communication dynamics reflect cognitive deficits in temporal lobe epilepsy. Epilepsia, 2021, 62(4): 1022–1033. doi: 10.1111/epi.16864 33705572

[56] Fovet T, Yger P, Lopes R, et al. Decoding activity in Broca’s area predicts the occurrence of auditory hallucinations across subjects. Biological psychiatry, 2022, 91(2): 194–201. doi: 10.1016/j.biopsych.2021.08.024 34742546

[57] Peterson C, Peterson J L, Webb J. Factors influencing the development of a theory of mind in blind children. British journal of developmental psychology, 2000, 18(3): 431–447. 10.1348/026151000165788.

[58] Green S, Pring L, Swettenham J. An investigation of first‐order false belief understanding of children with congenital profound visual impairment. British journal of developmental psychology, 2004, 22(1): 1–17. 10.1348/026151004772901087.

[59] Brambring M, Asbrock D. Validity of false belief tasks in blind children. Journal of autism and developmental disorders, 2010, 40(12): 1471–1484. doi: 10.1007/s10803-010-1002-2 20379770

[60] Keeler W R. Autistic patterns and defective communication in blind children with retrolental fibroplasia. Proceedings of the annual meeting of the American psychopathological association, 1956, 64–83. . 13518120

[61] Wing L. The handicaps of autistic children—a comparative study. Journal of child psychology and psychiatry, 1969, 10: 1–40. doi: 10.1111/j.1469-7610.1969.tb02066.x 4243575

[62] Brown R P, Hobson P, Lee A, et al. Are there “autistic-like” features in congenitally blind children? Journal of child psychology and psychiatry. 1997, 38(6): 693–703. doi: 10.1111/j.1469-7610.1997.tb01696.x 9315979

[63] Wrzesińska M, Kapias J, Nowakowska-Domagała K, et al. Visual impairment and traits of autism in children. Psychiatria Polska, 2017, 51(2): 349–358. 10.12740/pp/onlinefirst/61352. 28581542

[64] Lipton E L. Insights from the blind; comparative studies of blind and sighted infants. Journal of the American Psychoanalytic Association, 1979, 27(1): 273–276. 10.1176/ajp.135.5.630.

[65] Ryan J. Harvard Medical School scientists reverse age-related vision loss, eye damage from glaucoma in mice. [2020-12-2]. https://news.harvard.edu/gazette/story/2020/12/reversing-glaucoma-damage-and-vision-loss/.

[66] Collerton J, Davies K, Jagger C, et al. Health and disease in 85 years old: baseline findings from the Newcastle 85+ cohort study. British medical journal, 2009, 339: 1–11. 10.1136/bmj.e4462. PMC2797051 20028777

[67] Kelley J M, Kraft-Todd G, Schapira L, et al. The influence of the patient-clinician relationship on healthcare outcomes: a systematic review and meta-analysis of randomized controlled trials. Plos one, 2014, 9(4): e94207. doi: 10.1371/journal.pone.0094207 24718585 PMC3981763

