# Supplementary material for: Impacts of visual impairment on pragmatic impairment: A systematic review and meta-analysis
Source: PLoS One. 2023 Dec 8;18(12):e0294326. doi: 10.1371/journal.pone.0294326 (PMC10707542; doi:10.1371/journal.pone.0294326)
Supplement: S1 File — (PDF) [file pone.0294326.s002.pdf]

MeSH = 3:
Firstly, "vision, low"[MeSH Terms] OR "vision disorders"[MeSH Terms] OR visual impairment[Text Word] were used to search articles in MeSH.
Because pragmatic impairment can be replaced by these terms:
Development Disorder, Language
Disorder, Language Development
Disorders, Language Development
Language Development Disorder
Speech or Language, Developmental Disorder
Language Disorders, Developmental
Developmental Disorder, Speech or Language
Developmental Language Disorders
Developmental Language Disorder
Language Disorder, Developmental
Speech Delay
Delay, Speech
Delays, Speech
Speech Delays
Semantic-Pragmatic Disorder
Semantic Pragmatic Disorder
Semantic-Pragmatic Disorders
Auditory Processing Disorder, Central
Central Auditory Processing Disorder
Language Delay
Language Delays

Then, we combined different terms with the same meanings, for instance:
First, by (TS=(visual impairment)) AND TS=(pragmatic impairment), we acquired 58 articles. Then, by manual screening, we retained 31 articles.

The similar search steps were used in Psychinfo CNKI, Ovid, HMIC, EBSCO, ERIC, WOS and Google.

Finally, by manual screening, we acquired the following article quantity, respectively:
Psychinfo=87; CNKI=7; Ovid , HMIC,EBSCO, ERIC, WOS= 134, Google.com=1.
