# Supplementary material for: Impacts of visual impairment on pragmatic impairment: A systematic review and meta-analysis
Source: PLoS One. 2023 Dec 8;18(12):e0294326. doi: 10.1371/journal.pone.0294326 (PMC10707542; doi:10.1371/journal.pone.0294326)
Supplement: S2 File — (PDF) [file pone.0294326.s003.pdf]

PRISMA 2020 flow diagram for new systematic reviews which included searches of databases and registers only

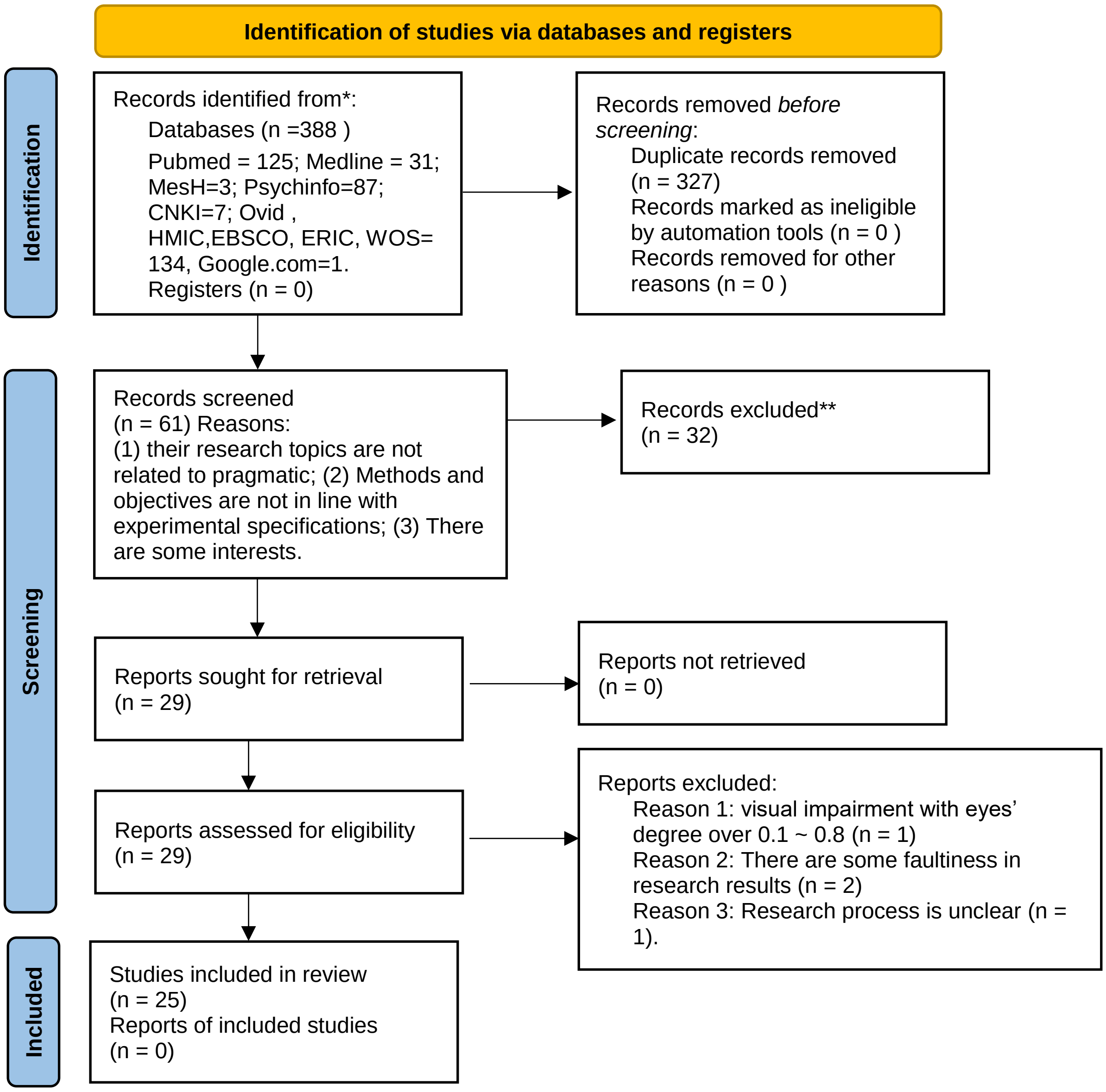

*Consider, if feasible to do so, reporting the number of records identified from each database or register searched (rather than the total number across all databases/registers).

**If automation tools were used, indicate how many records were excluded by a human and how many were excluded by automation tools.
